# Retrosternal Colonic Bypass for Intractable Esophagogastric Anastomotic Stricture Post-Ivor-Lewis Esophagectomy

**DOI:** 10.7759/cureus.67398

**Published:** 2024-08-21

**Authors:** Georgi Yankov, Magdalena Alexieva, Zaharinka Makshutova, Evgeni V Mekov

**Affiliations:** 1 Thoracic Surgery Department, University Hospital St. Ivan Rilski, Medical University of Sofia, Sofia, BGR; 2 Respiratory Diseases Department, University Hospital St. Ivan Rilski, Medical University of Sofia, Sofia, BGR

**Keywords:** complications, surgical treatment, gastroesophagoplasty, retrosternal bypass coloesophagoplasty, postoperative intractable stricture

## Abstract

Intractable stricture of the esophagogastric anastomosis after Ivor-Lewis esophagectomy for esophageal corrosive burn is an extremely rare complication. We present the case of a 28-year-old man with complaints of dysphagia and two attempts for bougie dilation without effect. Retrosternal colonic bypass and distal Roux-en-Y neoesophagojejunal reconstruction, as well as double enterostomy with Braun anastomosis, were performed.

## Introduction

Non-dilatable esophageal stricture after Ivor-Lewis gastroesophagoplasty for an esophageal corrosive burn is a rare complication requiring complex and precise surgical intervention with multiple anastomoses [1]. Surgical treatment is challenging and has the best long-term results. The surgical treatment of choice for these patients often involves an esophageal colonic bypass, where a segment of the colon is used to replace the diseased esophagus [2]. This method, combined with Roux-en-Y reconstruction of the neoesophagus using a segment of the jejunum, has shown favorable outcomes [3]. Selecting the left colonic artery as the feeding vessel for the neoesophagus and the retrosternal route for the bypass is critical to ensure adequate blood supply and minimize postoperative complications.

The following case report details the clinical presentation, diagnostic workup, and complex surgical management of a young male patient who developed a non-dilatable esophagogastric anastomotic stricture after an Ivor-Lewis esophagectomy performed for esophageal damage due to caustic ingestion.

## Case presentation

А 28-year-old man was admitted to the thoracic surgery department for the first time with complaints of dysphagia for five years after inadvertent ingestion of caustic soda. Later, a series of dilatations with Savary-Gilliard dilators were performed in a gastroenterology clinic with a temporary effect. Four years later, an Ivor-Lewis gastroesophagoplasty was performed in another institution, but after three months, the symptoms of dysphagia recur. According to the medical documentation, gastroesophagoplasty was performed through an upper median laparotomy and a right posterolateral thoracotomy, a classic Ivor-Lewis operation. A gastric tube was created and placed in the posterior mediastinum (posterior mediastinal root) with the gastroesophageal anastomosis performed just above the level of the azygos vein. In the following three months, two dilatations with dilators were performed in a gastroenterology clinic with a temporary effect, and the stricture was not passed during the next dilatation attempt.

**Figure 1:**
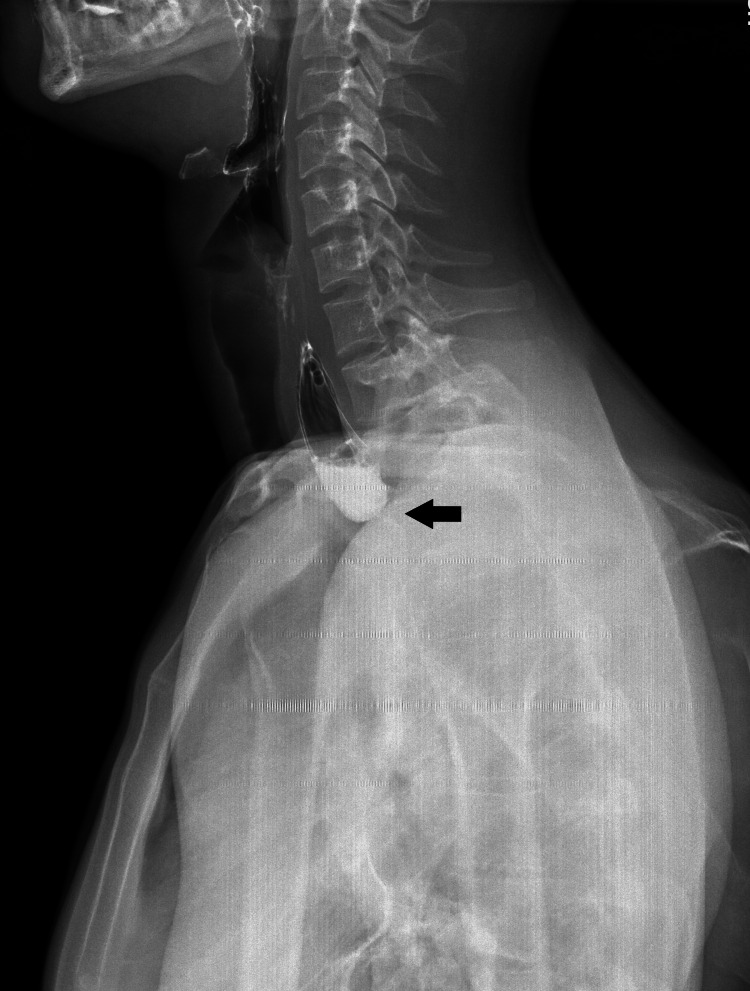
Contrast X-ray after Ivor-Lewis gastroesophagoplasty A visualization of the contrast medium stop at the transition between the neck and chest just above the previously performed Ivor-Lewis esophagogastric anastomosis and the localization of the stricture itself (black arrow)

Fibroesophagogastroscopy showed stricture in the area of the esophagogastric anastomosis with a diameter of 2 mm, located 18 cm from the dentition. In the esophageal contrast study, there is X-ray evidence of stricture of the cervicothoracic part of the esophagus (Figure 1).

**Figure 2:**
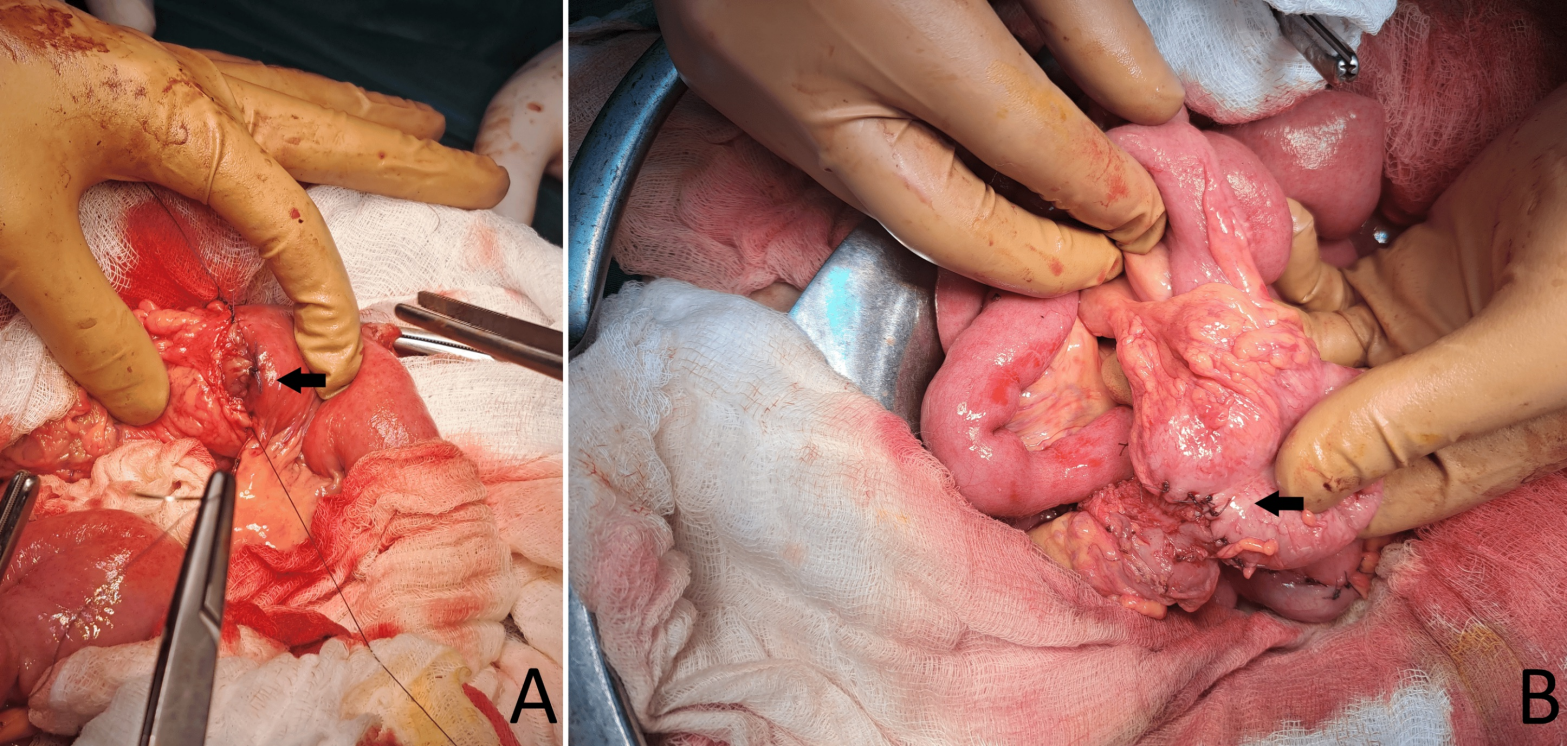
(A) End-to-end neoesophagojejunal anastomosis. (B) End-to-end ceco-descendo anastomosis

The abdomen was opened with upper median laparotomy continued into a lower one about 5 cm below the level of the umbilicus. Massive adhesions of the liver to the omentum, transverse colon, duodenum, pancreatic head, and anterior abdominal wall, due to previous surgery, necessitated total debridement and omentectomy. The entire colon from the cecum to the transition of the rectosigmoid colon is mobilized. A graft for a neoesophagus was prepared on a feeding vessel ascending branch of the left colic artery. The right colic artery, media colic artery, and left colic artery with their accompanying veins were transected and ligated. The colon was transected proximally just above the cecum and distally at the level of the distal part of the descending colon. However, the ileocolic artery was only dissected, but not transected. The reconstructed colonic blood flow was assessed by the indocyanine green (ICG) fluorescence method. A left neck incision was performed in front of the sternocleidomastoid muscle. A retrosternal route was prepared by sharp and blunt dissection. The tailored isoperistaltic neoesophagus was pulled up to the neck. A latero-lateral esophago-colonic anastomosis was performed just proximally above the stricture located at the level of the jugular notch of the sternum. Due to the completely intrathoracic location of the stomach, the small intestine was interrupted about 30 cm distal to the ligament of Treitz, and a Roux-en-Y neoesophagojejunal anastomosis was performed (Figure 2A). Colonic continuity was restored by end-to-end ceco-descendo anastomosis (Figure 2B). To decompress the gastrointestinal tract, a double jejunostomy with two 20-Fr catheters was performed 20 cm distal to the Roux-en-Y jejuno-jejunal anastomosis. One was guided in an oral direction to the antrum of the intrathoracically located stomach and the other in a distal direction into the lumen of the small intestine. The double jejunostomy was accomplished according to the Kader technique, and under that, a side-to-side Braun anastomosis was performed to prevent compromising the jejunal lumen and postoperative ileus phenomena. Due to a change in the topic of the appendix, an appendectomy was performed.

The catheter from the debarrassing enterostomy was removed on the eighth postoperative day and the feeding catheter, on the 20th postoperative day in outpatient settings. The patient was discharged on the 10th postoperative day, and six months after the operation, he is in excellent general condition and without any complaints.

## Discussion

Surgical site infections are the most common postoperative complication after colorectal and gastrointestinal surgery, causing pain and suffering to patients [4]. In addition, this complication has been associated with negative economic impact, increased morbidity, extended postoperative hospital stay, readmission, sepsis, and death. Strictures of the anastomosis could occur in 14% of patients after esophagectomy, and the main risk factors are chronic obstructive pulmonary disease (COPD), leakage, and anastomosis technique [5]. Postoperative esophageal strictures are best evaluated by esophagoscopy and contrast esophageal X-ray. Surgical treatment of severe corrosive strictures most often consists of colon replacement [1], and retrosternal interposition with the colon is the safest [6]. For caustic strictures, an isoperistaltic left colonic graft based on the left colic artery is the preferred technique [7]. We also favor the use of a left colon, both retrosternal and posterior mediastinal, through the route of the native esophagus when the latter is removed during the same operation. The technique for performing an anastomosis after esophageal resection continues to be debatable [8].

Local ischemia and tension of the anastomosis are the usual causes of postoperative stricture [9]. In the described case, resection of the esophagus and intrathoracic esophagogastric anastomosis were performed using a 25 mm circular stapler, and a stricture was formed in the anastomosis area three months later. In our opinion, this intervention was not the most appropriate, since usually in case of corrosive burns, abnormalities in the esophagus in most cases are already present in its proximal parts. Attempts to dilate with bougies have a short-term effect. Currently, reoperation is rarely required, but esophagocolostomy or a jejunal bypass graft may be performed [10]. Surgical treatment is safest and has the best long-term results. The operative intervention performed in our department was extremely difficult and challenging because of the massive adhesions and the altered anatomy from the previous intervention. Considering that the entire stomach was completely located in the mediastinum, we decided to perform a retrosternal bypass through an isoperistaltic neoesophagus of a nutrient vessel of the left colic artery, and the distal end of the neoesophagus was anastomosed with a jejunum by the Roux-en-Y technique. A double enterostomy was performed distal to jejuno-jejunal anastomosis for upper gastrointestinal decompression and early enteral feeding, and below it, we performed a Braun latero-lateral small bowel anastomosis. Colonic continuity was restored by end-to-end ceco-descendo anastomosis.

## Conclusions

Treatment of intractable and non-dilatable anastomotic stricture after gastroesophagoplasty for esophageal corrosive burn is challenging and extremely difficult. A safe technique is retrosternal colonic bypass and distal anastomosis of the neoesophagus with the jejunum according to the Roux-en-Y technique. Double enterostomy with Braun anastomosis for the decompression of the upper gastrointestinal tract and early enteral feeding is of paramount importance. For the prevention of this type of complication, the main requirement is to remove the entire damaged esophagus within healthy borders and to perform an atraumatic anastomosis with good blood supply without tension.
